# Decreased HIV-Specific T-Regulatory Responses Are Associated with Effective DC-Vaccine Induced Immunity

**DOI:** 10.1371/journal.ppat.1004752

**Published:** 2015-03-27

**Authors:** Vedran Brezar, Nicolas Ruffin, Laura Richert, Mathieu Surenaud, Christine Lacabaratz, Karolina Palucka, Rodolphe Thiébaut, Jacques Banchereau, Yves Levy, Nabila Seddiki

**Affiliations:** 1 Equipe 16, Inserm U955, Créteil, France; 2 Faculté de médecine, Université Paris Est, Créteil, France; 3 Vaccine Research Institute (VRI), Créteil, France; 4 Université Bordeaux, ISPED, Centre INSERM U897-Epidemiologie-Biostatistique, Bordeaux, France; 5 INSERM, ISPED, Centre INSERM U897-Epidemiologie-Biostatistique, Bordeaux, France; 6 CHU de Bordeaux, Pôle de santé publique, Bordeaux, France; 7 INRIA SISTM, Talence, France; 8 Ralph M. Steinman Center for Cancer Vaccines, Baylor Institute for Immunology Research, Baylor Research Institute, Dallas, Texas, United States of America; 9 The Jackson Laboratory for Genomic Medicine, Farmington, Connecticut, United States of America; 10 Service d'immunologie clinique et maladies infectieuses, AP-HP, Hôpital H. Mondor—A. Chenevier, Créteil, France; Vaccine Research Center, UNITED STATES

## Abstract

The role of regulatory T cells (Tregs) in vaccination has been poorly investigated. We have reported that vaccination with *ex vivo*-generated dendritic-cells (DC) loaded with HIV-lipopeptides (LIPO-5-DC vaccine) in HIV-infected patients was well tolerated and highly immunogenic. These responses and their relation to viral replication following analytical treatment interruption (ATI) were variable. Here, we investigated whether the presence of HIV-specific Tregs might explain these differences. Co-expression of CD25, CD134, CD39 and FoxP3 was used to delineate both antigen-specific Tregs and effectors T cells (Teffs). Median LIPO-5 specific-CD25^+^CD134^+^ polyfunctional T cells increased from 0.1% (IQR 0-0.3) before vaccination (week -4) to 2.1% (IQR 1.1-3.9) at week 16 following 4 immunizations (p=0.001) and were inversely correlated with maximum viral load following ATI (r=-0.77, p=0.001). Vaccinees who displayed lower levels of HIV-specific CD4^+^CD134^+^CD25^+^CD39^+^FoxP3^+^ Tregs responded better to the LIPO-5-DC vaccine. After vaccination, the frequency of HIV-specific Tregs decreased (from 69.3 at week -4 to 31.7% at week 16) and inversely correlated with HIV-specific IFN-γ-producing cells (r=-0.64, p=0.002). We show that therapeutic immunization skewed the HIV-specific response from regulatory to effector phenotype which impacts on the magnitude of viral replication following ATI.

## Author Summary

Highly active antiretroviral therapy (HAART) has considerably decreased AIDS-related mortality and morbidity in recent years. Nevertheless, the search for effective vaccine to combat HIV is in the limelight of modern medical research. In clinical trial settings, T-cell responses are routinely measured following vaccinations. However, the measurement of antigen-specific regulatory T-cell (Tregs) responses is omitted most of the time, since their detection is not possible with the use of standard assays. Following a phase I clinical trial in which autologous dendritic-cells pulsed with HIV-lipopeptides were used to induce T-cell responses, we used a novel assay to detect a whole range of T-helper responses, including Tregs. We report very high levels of HIV-specific Tregs responses in infected patients and interestingly, we observed that the dendritic cell-based vaccine shifted the responses from regulatory to effector phenotype, which impact on the magnitude of viral rebound after treatment interruption.

## Introduction

AIDS-related mortality and morbidity have decreased considerably since the introduction of highly active antiretroviral therapy (HAART). Yet, HIV infection cannot be eradicated and lifelong HAART treatment is associated with several co-morbidities [1–3]. It is currently thought that the control of the HIV-1 epidemic will require both prophylactic and therapeutic vaccines. Despite considerable investments, potent HIV vaccines are not yet available [4,5]. Prophylactic vaccine development had mainly been focused on the induction of neutralizing humoral responses [6]. Several studies conducted in HIV-infected individuals or in Non-Human Primates have shown that vaccines which could induce HIV-specific T-cell responses may be effective against HIV replication [6–10].

Monocyte-derived dendritic cells (moDCs) pulsed *ex vivo* with tumor- or pathogen-derived antigens can induce T-cell responses in animal models [11,12]. This strategy has been used in the context of HIV infection in several studies [13,14]. We and others [15–18] have shown that DC-based vaccines were safe and efficient in inducing HIV-specific immune responses. *Ex vivo* generated autologous DCs loaded with HIV-derived long lipopeptides covering gag, nef and pol epitopes (LIPO-5-DC vaccine) were immunogenic *in vivo*. The induced polyfunctional HIV-specific responses were negatively correlated with the maximum viral load after HAART cessation [18]. In the present study, we have extended the characterization of vaccine-elicited T-cell responses to regulatory T-cell (Tregs) responses. Induction of Tregs by an HIV-vaccine is not a desired outcome as these cells can suppress HIV-specific effector T-cells (Teffs) responses [19].

Current assays used to evaluate antigen-specific responses, including effector cytokine or proliferative capacity measurements, are limited as they do not take into account antigen-specific Tregs because these cells are known to be anergic *in vitro* [20]. Moreover, detection of antigen-specific CD4^+^ T-cell responses by cytokine production (intracellular staining) after exposure to antigen can be misleading since the kinetics of cytokines secretion such as IFN-γ, IL-17, IL-2 or IL-10, is very variable. Therefore, we used here the “OX40 assay” [21] to simultaneously detect a full range of Th responses including antigen-specific Tregs responses [22]. CD134 (OX40) is an inducible co-stimulatory molecule from the TNFR superfamily. It is expressed on recently activated T cells and its interactions with its ligand promote survival, proliferation as well as cytokine production [23]. The coexpression of CD134 and CD25 along with Tregs-specific markers, FoxP3 and CD39, allowed the detection of both HIV-specific Tregs and cytokine-producing Teffs.

We report that HIV-infected individuals harbor high levels of HIV-specific Tregs at baseline. The LIPO-5-DC vaccine preferentially induces Teffs responses and shifts the HIV-specific Tregs:Teffs ratio towards polyfunctional effector responses that inversely correlate with maximum viral load rebound after treatment interruption. Interestingly, vaccinees who display lower levels of HIV-specific CD4^+^CD134^+^CD25^+^CD39^+^FoxP3^+^ Tregs, show better Teffs responses to the LIPO-5-DC vaccine.

## Results

### HIV-specific CD4^+^ T-cell responses are induced upon vaccination with autologous moDCs loaded with LIPO-5 vaccine

Nineteen HIV-1 infected individuals under successful antiretroviral therapy have been included in this pilot study (Table 1) out of which we had access to frozen samples of 14 participants. Patients received LIPO-5-DC vaccine every 4 weeks during 16 week period. Blood was drawn 4 weeks prior to first vaccination (week -4) and 4 weeks after the last (week 16). Virological endpoints following analytical treatment interruption (ATI) starting at week 24, were defined at the study entry due to safety issues. Primary endpoint was the maximum viral load while predefined secondary virological endpoints were the time to viral rebound, the area under the curve of viral load, and the slope of the initial viral rebound [18].

**Table 1:** Patients’ characteristics.

Characteristics	All trial participants (n = 19)		Participants included in sub-study (n = 14)	
	Median (IQR)	N (%)	Median (IQR)	N (%)
**Male**		16 (84)		12 (86)
**Age (years)**	44 (35–49)		45 (35–51)	
**Body Mass Index (kg/m** ^**2**^)	27 (25–29)		27 (25–28)	
**HIV clinical stage A**		17 (89)		13 (93)
**HIV clinical stage B**		2 (11)		1 (7)
**Nadir CD4** ^**+**^ (**/mm** ^**3**^)	355 (319–452)		334 (316–370)	
**CD4** ^**+**^ **at wk -8 (/mm** ^**3**^)	712 (628–961)		697 (628–898)	
**CD4** ^**+**^ **at wk0 (/mm** ^**3**^)	670 (553–832)		647 (536–756)	
**HAART at wk -8 /NRTI**		19 (100)		14 (100)
**HAART at wk -8 /NNRTI**		17 (89)		12 (86)
**HAART at wk -8 /PI**		3 (16)		3 (21)
**Time (years) between the start of the first HAART and inclusion**	8.6 (5.4–12.6)		9.0 (5.4–11.8)	
**Time (years) between the start of the current HAART and inclusion**	2.6 (1.3–3.7)		2.7 (0.8–3.7)	

We first determined both frequency and phenotype of CD4^+^ and CD8^+^ T-cell subsets *ex-vivo* to verify whether the vaccine influenced these parameters. A slight, although statistically significant increase in the CD4^+^/CD8^+^ T-cell ratio after vaccination (week 16) was observed (Table 2). No changes in CD8^+^ Tregs percentages or in activation (CD38/HLADR) and/or exhaustion (PD-1/2B4/Blimp-1) markers within the CD4^+^ and CD8^+^ T-cell compartments were found. Bulk CD4^+^CD25^+^CD127^low^ Tregs fraction increased slightly after vaccination probably reflecting the increase in CD4^+^ T-cell compartment (Table 2).

**Table 2:** Ex-vivo phenotype.

	week -4	week 16	p*
**%CD4**	**41.95** (38.18–52.18)	**45.25** (40.88–55.25)	**0.002**
**%CD8**	**47.15** (40.35–51.85)	**44.55** (37.48–48.63)	**0.001**
**CD4/CD8 ratio**	**0.8595** (0.7483–1.238)	**1.046** (0.8253–1.449)	**0.001**
**%Tregs**	**6.940** (5.478–8.298)	**7.345** (5.968–8.193)	**0.029**

Percentages of CD4^+^, CD8^+^ T cells, CD4^+^/CD8^+^ T-cells ratios as well as the percentages of CD4^+^CD25^hi^CD127^lo^FoxP3^+^ Tregs (among CD4^+^ T cells) at week -4 (prior vaccination) and week 16 (post vaccination). Medians with IQR values are indicated. P values are calculated based on Wilcoxon matched-pairs signed rank test. All cell staining and flow cytometry analysis was performed on freshly thawed samples from n = 14 HIV-1 infected individuals included in this study.

**Fig 1:**
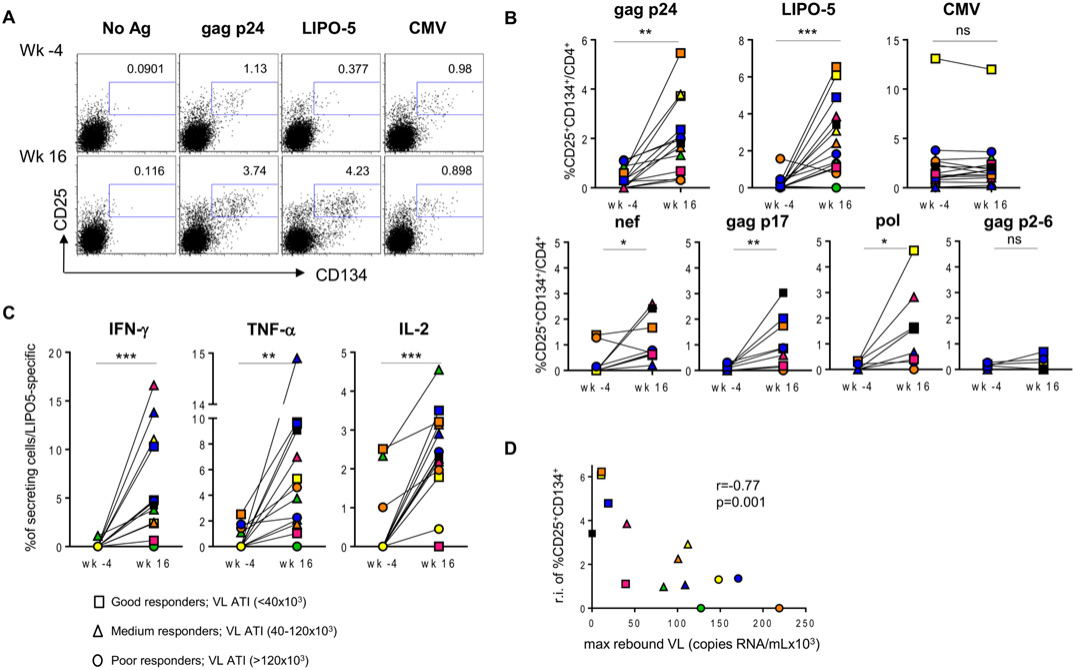
HIV-specific responses are significantly upregulated after the vaccination with LIPO-5-DC vaccine. Each patient is designated with its individual color/symbol code (refer to S1 Table). Symbols are attributed based on maximum viral load rebound after treatment interruption as indicated in the figure. (A) Representative plots for a single patient prior (wk -4) and after (wk 16) the vaccination. Cells were stimulated with gag p24 pool, LIPO-5 vaccine or CMV lysate as a control, stained and analyzed by flow cytometry 44 hours later. Plots show viable CD4^+^ T cells. (B) Graphs show the percentages of antigen-specific cells (CD134^+^CD25^+^) among CD4^+^ T cells after the stimulation with the indicated antigens (n = 14). (C) IFN-γ, TNF-α and IL-2 production among LIPO-5- specific cells (CD134^+^CD25^+^) (n = 14). (D) Graph shows the correlation between the relative increase in LIPO-5-specific response (response after the vaccination-response before vaccination) and maximal viral load rebound after HAART interruption (n = 14). Data were analyzed by Wilcoxon matched-pairs signed rank test. *p < 0.05; **p < 0.01; ***p < 0.001. Spearman coefficient is indicated (r) as well as p value.

We stratified (using symbols- square, triangle and circle) the patients according to the magnitude of maximum viral rebound following ATI. Thus, patients with good (squares), intermediate (triangles) and poor (circles) virological responses were defined according to the maximum viral load post-ATI (VL ATI <40x10^3^, 40x10^3^ <VL ATI <120x10^3^ and VL ATI >120x10^3^ copies/ml respectively). The three subgroups correspond to the tertiles of the VL distribution. We then compared the levels of antigen-specific CD4^+^ T cells measured using the “OX40 assay”, between these patient groups. PBMCs from before and after vaccination were stimulated with either HIV-derived peptide pools (gag p24), LIPO-5 vaccine (which is a pool of 5 lipopeptides, 2 gag, 2 nef and 1 pol) or CMV lysate for 44-hrs *in vitro*. A significant increase in both LIPO-5- and gag p24- specific responses (CD4^+^CD25^+^CD134^+^ cells) after vaccination was observed, while the responses to CMV remained unchanged (Fig. 1A-B upper panel). Good virological responders showed the greatest increase in immune responses (Fig. 1A-D). To check whether vaccine-induced immune responses and post-ATI viral load, was not driven by pre-HAART viral load levels, we performed additional analysis using the historical viral loads prior to any HAART. These analyses showed that the maximum viral load post-ATI in the trial was not associated with patient’s pre-HAART viral load (r = -0.03, p = 0.93).

The increase in activated LIPO-5-specific CD4^+^ T cells was accompanied by an increase in the frequency of cells expressing intracellular IFN-γ, TNF-α and IL-2 (Fig. 1C). Similar increases of cytokine-secreting cells were observed when gag p24, but not CMV (S1 Fig), was used as eliciting antigen. In 9 out of 14 patients from whom sufficient cell numbers were available, we confirmed the results by additional testing of HIV-peptide pools representing each of the individual immunogens in the LIPO-5 vaccine. Interestingly, there was a significant increase in pol-, nef- or gag p17-specific responses (CD4^+^CD25^+^CD134^+^) but not to gag p2-6 (Fig. 1B, lower panel) that was not contained in the LIPO-5 vaccine. The specificity of the CD4^+^CD25^+^CD134^+^ T cells was further demonstrated by the co-expression of CD154, a marker of recently-activated antigen-specific cells [24] (S2 Fig).

### LIPO5-DCs induced strong polyfunctional CD4^+^ T-cell responses

Antigen-specific CD4^+^CD25^+^CD134^+^ cells are heterogeneous and express a wide range of transcription factors such as Tbx21, Gata3, Rorc, Foxp3 and Bcl-6 [25]. They comprise Th1-like cells that are commonly measured in standard ICS protocols but also other Th subtypes. To evaluate the functional profile of HIV-specific responses, we measured by Luminex the cytokines in the supernatants collected from the “OX40 assays” described above (44-hrs post-culture). Increases in IFN-γ, IL-2, IL-4, IL-21, IL-17F, TNF-α, MIP-1β, IL-3, IL-5, IL-9, IL-10, IL-13, IL-27 and sCD40L (S3A Fig) were observed after the vaccination. Notably, the increased levels of cytokines correlated with the increase of antigen-specific CD4^+^CD25^+^CD134^+^ T cells, thus indicating their polyfunctionality (S3B Fig). Moreover, we calculated multivariate immune scores (see Statistical analysis in Methods) to summarize the data across several immune markers. Based on cytokine-producing CD4^+^CD25^+^CD134^+^ T cells as well as IFN-γ, IL-2, IL-13 and IL-21 secretion assessed by Luminex, the median immune score increased significantly from median -6 (IQR -10 to -4) to 9 (IQR 9 to 10) between baseline and the post-vaccination time point (p = 0.008). Consistent with our previous report [18], the post-vaccination immune score showed a significant negative correlation with the maximum viral load after ATI (r = -0.79; p = 0.010).

**Fig 2:**
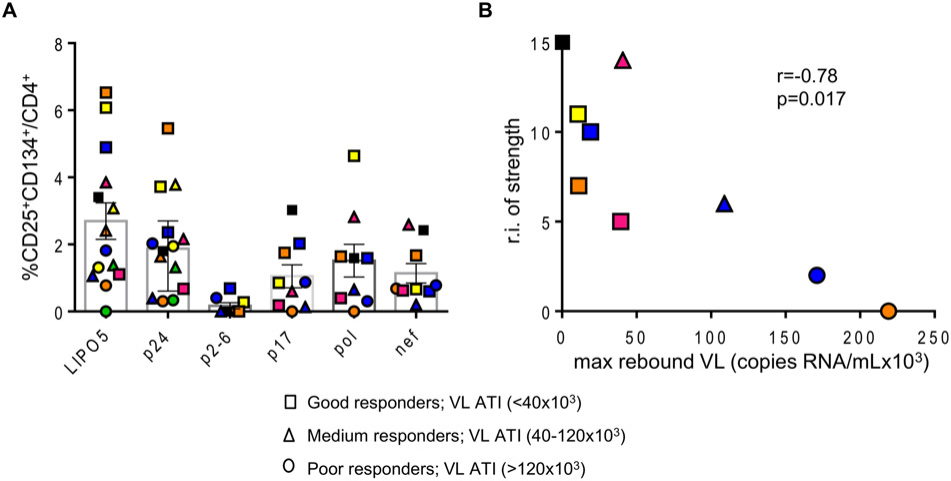
Overall strength of the HIV-specific responses is inversely correlated with maximum viral load after ATI. (A) Graph showing the responses for all individual patients to indicated antigens. (B) Correlation between the relative increase in the strength of the response (sum of the strengths of the response post vaccination–sum of the strengths of the response prior to vaccination) and maximal viral load rebound after HAART interruption (n = 9). Data were analyzed by Wilcoxon matched-pairs signed rank test. *p < 0.05; **p < 0.01; ***p < 0.001. Spearman coefficient is indicated (r) as well as p value.

In addition, the relative increases in LIPO-5-specific cells inversely correlated with the maximum observed viral load rebound after ATI (Fig. 1D). As mentioned above, in this phase I trial, the follow up post-ATI was limited to a duration of 24 weeks (from wk24 to wk48) to ensure participants’ safety, therefore several patients did not reach stable levels of viral load within this short period. In order to verify our observations reported in Fig. 1D, we used average viral load levels after ATI (S4A-B Fig) as well as viral loads observed at the end of the follow up (week 48, 6 months post ATI except for two patients who resumed HAART prior to that time point, S4C Fig) and we have reached the same conclusions. The good virological responders (low maximum viral load after ATI) displayed the highest specific CD4^+^CD25^+^CD134^+^ T-cell responses. Similar inverse correlation was observed with gag p24 (S5 Fig), though the correlation was stronger for LIPO-5 than for gag p24 (r = -0.77, p = 0.001 for LIPO-5 vs r = -0.60, p = 0.026 for gag p24 pool). This suggests that the responses covering more than 1 peptide pool (breadth) might be more predictive of vaccine efficacy outcome. The graph showing the frequency of CD25^+^CD134^+^ T-cell specific responses for each peptide pool and for each patient, reveals that the good virological responders responded to more than one peptide pool, suggesting that vaccine efficacy is linked to the breadth of the response (Fig. 2A). Our functional assay allows us to further determine the strength of the HIV-specific responses. We gave empirical scores to the antigen-specific responses for each peptide pool from 1 to 4 based on the percentages of CD4^+^CD25^+^CD134^+^ antigen-specific cells measured at week 16 post-vaccination (S1 Table). Importantly, the overall strength of the response inversely correlated with the maximum of viral load after ATI (r = -0.78, p = 0.017) (Fig. 2B). In addition, patient N19 (black square), who did not experience viral rebound after ATI, showed the highest combination of breadth and strength of HIV-specific responses (S1 Table).

These data underline that LIPO-5-DC vaccination elicited a robust polyfunctional T-cell response which relies on both strength and breadth of the responses, a feature commonly desired for a functional HIV vaccine.

### CD25^+^CD134^+^CD39^+^FoxP3^+^ Tregs are part of the HIV-specific response, originate from CD25^hi^ T cells and are suppressive *in vitro*

Antigen-specific CD4^+^ T cells include both CD25^+^CD134^+^CD39^+^FoxP3^+^ Tregs and CD25^+^CD134^+^CD39^-^FoxP3^-^ Teffs that can produce IFN-γ, TNF-α and IL-2 (S6A Fig). CD25^+^ cells that have not upregulated CD134 post 44hrs stimulation, include ~90% of FoxP3^+^ positive cells. These cells produce no or very little IFN γ, TNF-α or IL-2 (S6B Fig).

**Fig 3:**
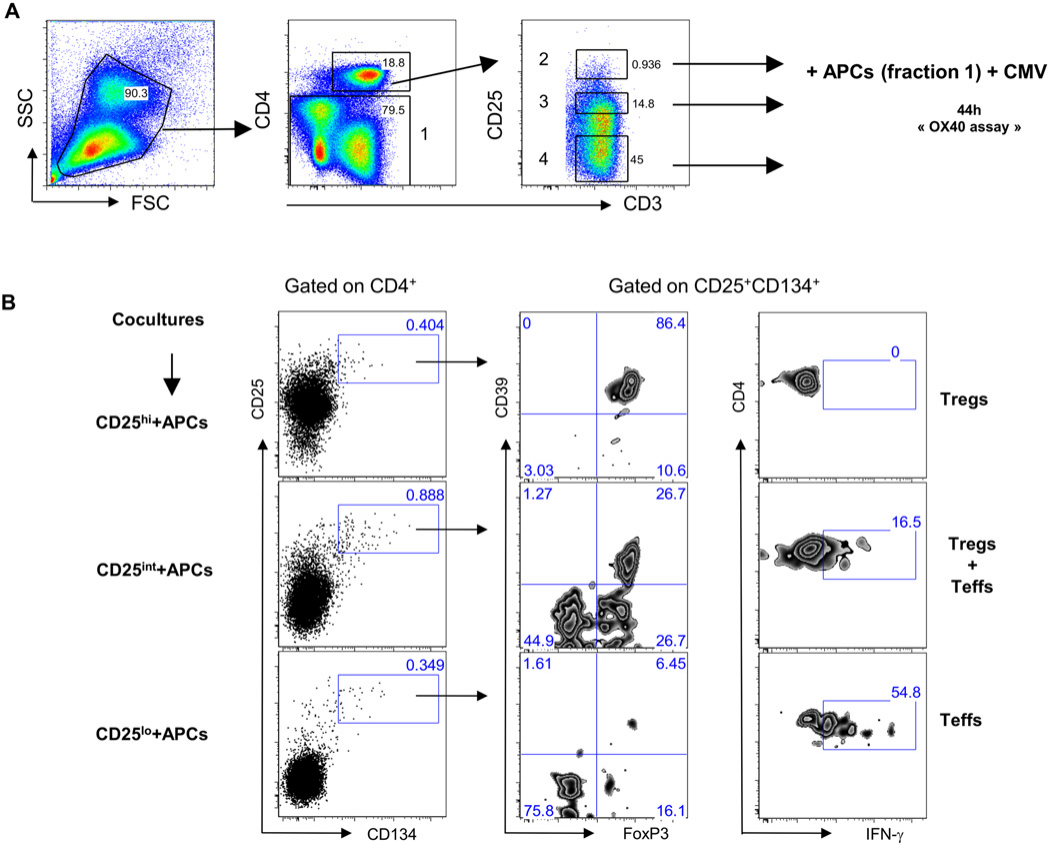
Antigen-specific Tregs originate from CD25^hi^ cells. (A) Plots showing the sorting strategy for CD4^+^CD25^hi^, CD4^+^CD25^int^ and CD4^+^CD25^lo^ populations as well as CD4^neg^. (B) Pre-sorted CD25 high, intermediate or low (left side) fraction were mixed with CD4^neg^ cells and stimulated for 44h with CMV lysate. Gating strategy is given for each fraction. Antigen-specific Tregs (CD39^+^FoxP3^+^IFN-γ^-^) originate from CD25^hi^ fraction while Teffs (CD39^-^FoxP3^-^IFN-γ^+^) originate from CD25^lo^ fraction. The figure is representative of 3 individual experiments.

**Fig 4:**
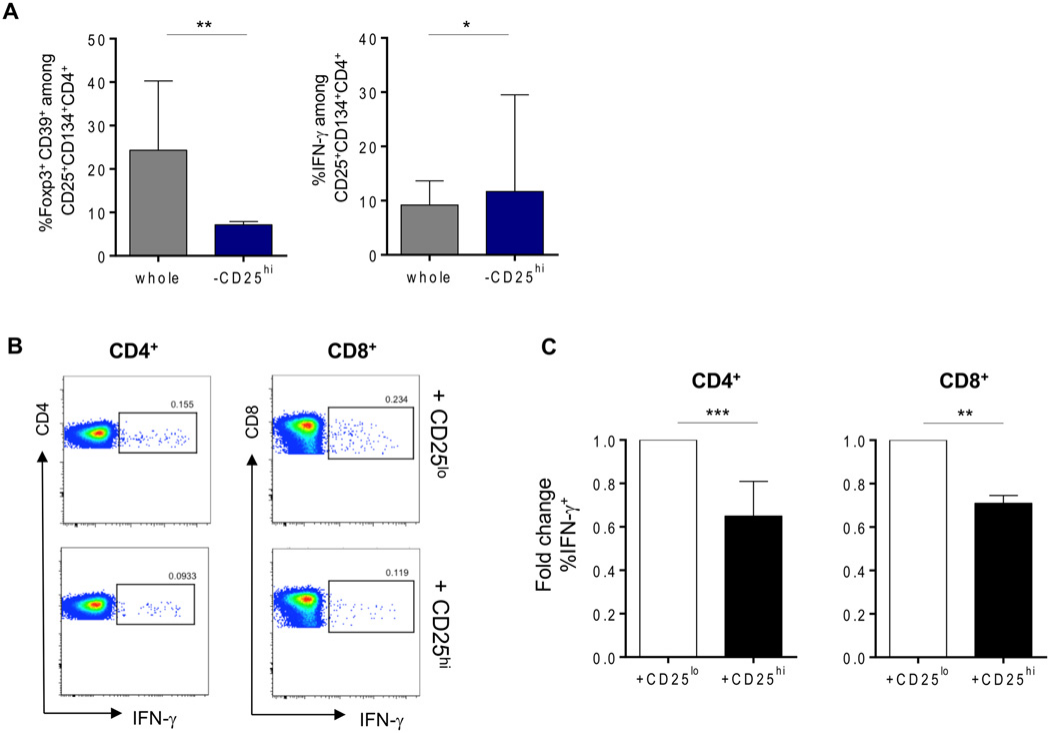
Tregs can suppress HIV-specific responses in vitro. (A) Percentages of gag p24-specific Tregs (CD134^+^CD25^+^CD39^+^FoxP3^+^) or IFN-γ-producing cells after stimulation in whole or Tregs-depleted fractions. (B) Representative plots of suppression assays in which depleted Tregs from (A) (lower panel) or CD25^lo^ fraction (upper panel) were cocultured in 1:2 ratio with CFSE-labeled PBMCs in overnight culture in the presence of 2μg/mL of gag p24 peptide pool, 1μg/mL of αCD28 and αCD49d and 10μg/mL of Brefeldin A. (C) Graphs showing percent of both CD4^+^ and CD8^+^ IFN- γ-secretion suppression (n = 9). Data were analyzed by Wilcoxon matched-pairs signed rank test. *p < 0.05; **p < 0.01; ***p < 0.001.

We sought to determine the origin of the two antigen-specific CD4^+^CD25^+^CD134^+^CD39^+^FoxP3^+^ Tregs and CD4^+^CD25^+^CD134^+^CD39^-^FoxP3^-^ Teffs subsets. CD4^+^ T cells were sorted based on their high, intermediate or low expression of CD25 (gating strategy on Fig. 3A) and then mixed with CD4^neg^ cells (fraction 1 that includes all cells that are outside the CD4 T-cells gate) at 1:4 ratio. We used CMV lysate to stimulate the cells. Forty-four hours later, cells were stained for IFN-γ, FoxP3 and CD39. The results in Fig. 3B show that antigen-specific CD4^+^CD25^+^CD134^+^CD39^+^FoxP3^+^ Tregs originated from CD25^hi^ cells that upregulated CD134 upon stimulation. These cells did not produce IFN-γ (Fig. 3B, right panel). In contrast, CD4^+^CD25^+^CD134^+^CD39^-^FoxP3^-^ Teffs, secreting high levels of IFN-γ, originated from CD25^lo^ cells. Finally, cells expressing intermediate levels of CD25 prior stimulation contained a mixture of antigen-specific Tregs and Teffs (Fig. 3B). To check whether CD4^+^CD25^+^CD134^+^CD39^+^FoxP3^+^ Tregs are thymically derived or induced in the periphery, we included an anti-Helios monoclonal antibody in our experiments. This molecule was recently proposed as a marker of thymically derived Tregs [26], although these studies are still quite controversial [27]. We observed that CD39^+^ Tregs, regardless of their antigen specificity, are Helios^+^ suggesting they might be of thymic origin (S7 Fig). This observation surely needs confirmation since more reliable markers will hopefully be available in the future. To fully define CD4^+^CD25^+^CD134^+^CD39^+^FoxP3^+^ cells as Tregs, we performed functional assays [28–30]. Depleting CD25^hi^ Tregs [31] prior to stimulation led to an increase in antigen-specific IFN-γ-producing cells (Fig. 4A right panel) and a decrease in CD4^+^CD25^+^CD134^+^CD39^+^FoxP3^+^ T cells (Fig. 4A left panel). These results confirm that antigen-specific Tregs originate from CD25^hi^ Tregs. As shown in Fig. 4B and C, CD25^hi^ but not CD25^lo^ cells suppressed CD4^+^ and CD8^+^ IFN-γ and TNF-α responses (ratio 1:2, Tregs:Teffs) after *in vitro* stimulation with a pool of gag p24 peptides. Due to the scarcity of the isolated Tregs, we could not test higher ratios (1:1, Tregs:Teffs), which can explain lower levels of suppression (30–35%) we detected in our experiments (Fig. 4C). As previously shown [32], likely a Treg:Teffs ratio of 1:1 would show a higher suppressive activity.

### HIV-specific CD25^+^CD134^+^CD39^+^FoxP3^+^ Tregs responses decrease after the vaccination

To investigate the influence of Tregs on the LIPO-5-DC-induced responses, we measured antigen-specific CD4^+^CD25^+^CD134^+^CD39^+^FoxP3^+^ Tregs in patients’ peripheral blood prior to and after vaccination.

**Fig 5:**
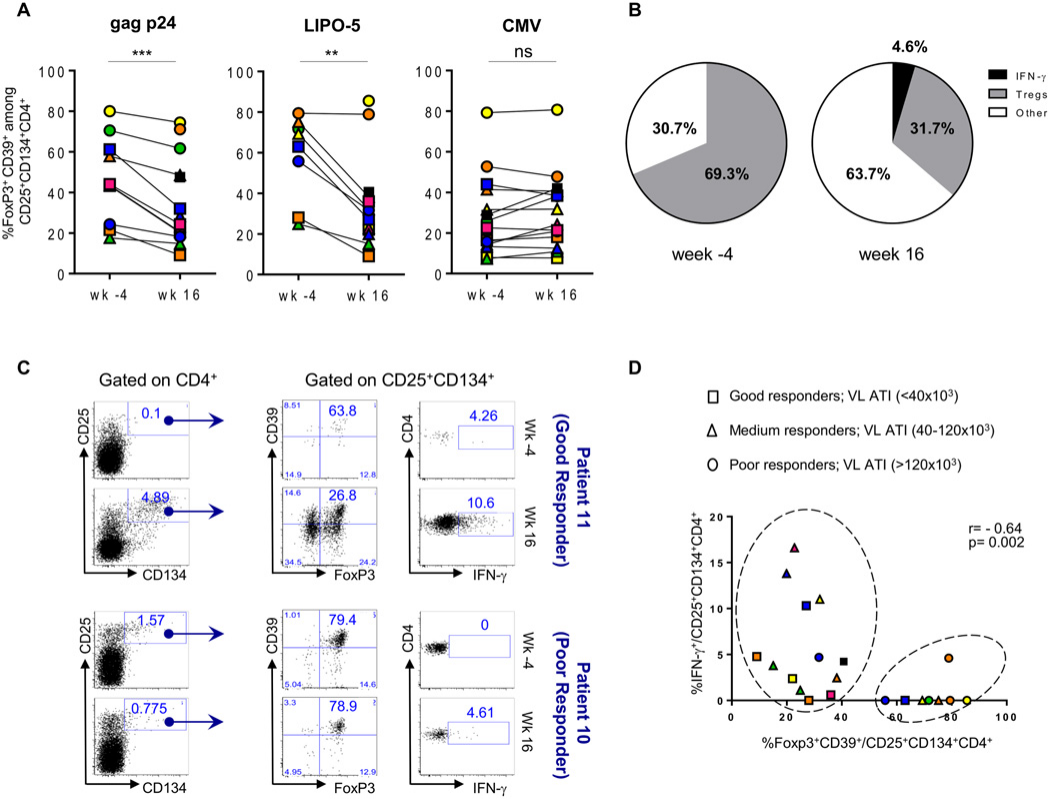
HIV-specific Tregs/Teffs ratio is affected by vaccination. (A) Gag p24-, LIPO-5- and CMV-Tregs responses prior and after vaccination (n = 14). (B) Pie chart showing LIPO-5-specific Tregs among LIPO-5-specific (CD134^+^CD25^+^) cells prior and after vaccination (n = 14). (C) Representative profiles after in vitro stimulation with LIPO-5 showing good and poor responder to vaccination (based on a viral load rebound). (D) Correlation between IFN-γ-producing and Tregs cells among LIPO-5-specific cells after in vitro stimulation. Data were analyzed by Wilcoxon matched-pairs signed rank test. *p < 0.05; **p < 0.01; ***p < 0.001. Spearman coefficient is indicated (r) as well as p value.

The frequency of HIV-specific Tregs prior to vaccination was elevated, accounting for a median of 43.8% (IQR 24.3–61.2) of gag p24- and 69.3% (IQR 55.8–75.2) of LIPO-5-specific response (Fig. 5A). CMV-specific Tregs in the same patients accounted for 24.2% (IQR 14.3–41.4) of the total CMV-specific CD4^+^ T-cell response.

Following vaccination, proportions of HIV-specific Tregs significantly decreased (26.3% (IQR 20.2–48.5), p = 0.002, of gag p24- and 31.7% (IQR 22.1–38.2), p = 0.008, of LIPO-5-specific CD4^+^ T cells) and this was accompanied by an increase in IFN- γ-producing HIV-specific CD134^+^CD25^+^ CD4^+^ T cells: from median 0.0% to 5.6% (p = 0.009) among gag p24-specific and from median 0.0% to 4.6% (p = 0.001) among LIPO-5-specific CD4+ T cells. Thus, while Tregs responses were dominant (69.3%) over Teffs (30.7%) before vaccination (Fig. 5B), the balance shifted after vaccination and the proportion of Tregs decreased (31.7%) simultaneously with an increase in both IFN- γ-producing cells (4.6%) and in “other responses” (63.7%). These “other responses” that we have not determined yet are probably associated (directly or indirectly) with the significant production of IL-2, IL-4, IL-13, IL-17F, TNF-α, MIP-1β, IL-3, IL-5, IL-9, IL-10, IL-21, IL-27 and sCD40L, as measured in bulk PBMCs using Luminex technology (S3A-B Fig).

When patients were stratified according to the magnitude of maximum viral rebound following ATI, good ATI-responders showed decreased HIV-specific Tregs responses after vaccination as compared to poor ATI-responders (Fig. 5C-D). Fig. 5C (upper left and middle panels) illustrates the change in the flow cytometry plots from a representative good ATI responder (patient 11) showing the decrease in frequency of CD39^+^FoxP3^+^ specific Tregs within the CD134^+^CD25^+^ cells. In contrast, the lower panels (left and middle) illustrates the lack of change in the high frequency of CD39^+^FoxP3^+^ specific Tregs within the CD134^+^CD25^+^ cells from a representative poor ATI responder (patient 10). Right upper and lower panels in Fig. 5C show LIPO-5 specific IFN-γ responses for both patients. When these parameters were combined for all patients, we could see that majority of patients with high specific Tregs frequency and low IFN- γ levels are mainly poor ATI-responders (circles) and can be clustered together in Fig. 5D (right circle). Patients with low Tregs-specific responses (<40%) included mainly good and medium virological responders (squares and triangles in left circle, Fig. 5D) and showed medium to high IFN-γ responses (> 1%). Finally, CMV-specific responses, including Tregs and IFN-γ-producing cells, were unchanged before and after vaccination (Fig. 5A and S1 Fig).

**Fig 6:**
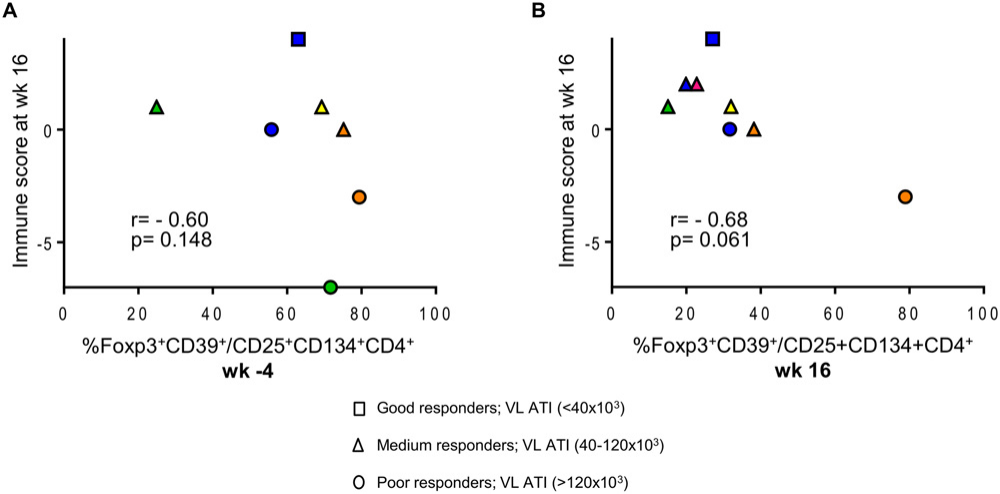
Patients with lower HIV-specific Tregs respond better to vaccination. (A) Correlation between the proportions of LIPO-5-specific Tregs among LIPO-5-specific CD4^+^ T cells at the baseline (wk -4) and immune score at wk 16. (B) Correlation between the proportions of LIPO-5-specific Tregs among LIPO-5-specific CD4^+^ T cells at the wk 16 and immune score at wk 16. Spearman coefficient for each correlation is indicated (r) as well as p value.

We explored further the data and used the multivariate immune score (See Statistical Analysis in Methods) to assess correlations between CD39^+^FoxP3^+^ LIPO-5-specific Tregs and effector functions after vaccination. Although this did not reach statistical significance likely due to the small sample size and limited statistical power, we found a consistent signal for a negative correlation between baseline Tregs and post-vaccination immune score (Fig. 6A), as well as between Tregs after vaccination and the immune score (Fig. 6B).

Together, these data suggest that the low IFN-γ responses usually found in HIV^+^ patients might be due to the presence of high percentages of HIV-specific Tregs among HIV-specific cells that might not be detected with current assays.

## Discussion

Efficient vaccines are characterized by the establishment of long-lived immunity. CD4^+^ T cells play an important role and are necessary for the control of viremia either directly or by providing help to B and CD8^+^ T cells [33,34]. CD4^+^ T cells comprise diverse populations, namely Th1, Th2, Th17, Tregs, Tfh and probably others [35]. We and others have previously shown that DC-based vaccines for HIV are feasible, safe, and well tolerated [17,18]. Our vaccine induced polyfunctional CD4^+^ and CD8^+^ T-cell responses, with a more prominent CD4^+^ response, that resulted in partial control of the viral load [18]. We also observed an inverse correlation between HIV RNA values after HAART interruption and frequencies of polyfunctional HIV-specific CD4^+^ T-cell responses detected 16 weeks after the start of vaccination protocol. One of the caveats of our study design is the fact that safety requirements for this phase I trial did not allow longer follow up periods after ATI. This resulted in the fact that more reliable measurement of post-ATI viral load rebound, such as viral load setpoint could not be clearly established. Therefore, we decided (consensus meeting with experts) to use maximum viral load rebound as a primary virological endpoint. This parameter is considered to be relevant as it reflects the capacity of the immune responses to control viral replication. Also, to strengthen our findings, we show that the average viral load post-ATI, as well as the viral load at the end of the follow up, inversely correlates with vaccine elicited CD4^+^ T-cell responses. However, these findings will be further corroborated in phase II trial (DALIA II), in which we will further address the effectiveness of the vaccine.

In this study, we explored in depth the frequency and function of antigen-specific CD4^+^ T-cell responses that were induced by the vaccine using the “OX40 assay” that allows the measurement of a whole range of antigen-specific cells regardless of their functional profile. Notably, this assay is very useful as it is able to detect HIV-specific CD4^+^ Tregs along with Teffs [21,22].

The role of Tregs in HIV infection has been extensively studied [36]. These cells may play a dual role firstly by decreasing immune activation, which is beneficial for HIV-infected individuals, but also secondly by suppressing anti-HIV responses. Even though the induction of Tregs was assessed in cancer [37,38] as well as in HIV vaccine trials [39], the induction of HIV-specific Tregs following vaccination has not been studied before. Indeed, the lack of tools that one can easily use in clinical trials setting has been preventing the measurement of Tregs-specific responses. Angin et al., recently reported the presence of gag-specific Tregs in infected individuals [40] by using MHC Class II tetramer loaded with gag peptide. Although interesting, this approach is challenging in clinical trials due to the genetic variability of MHC Class II as well as the limited availability of Class II tetramers. Tregs could also have different affinity with MHC comparing to Teffs, which could lead to differential staining and probable under- or over- estimation of their frequencies.

We were able to circumvent all these issues by the use of the inductive expression of CD134 on antigen-specific Tregs following an *in vitro* stimulation. Using this approach, the first surprising observation was that, prior to vaccination, a large proportion of HIV-specific Tregs with an activated phenotype (CD4^+^CD25^+^CD134^+^CD39^+^FoxP3^+^) were found. Forty-four percent of gag p24- and 69.3% of LIPO-5-specific CD4^+^ T cells were Tregs, as compared to 24.2% of CMV-specific CD4^+^ T-cell response. Whether these high proportions of Tregs among antigen-specific cells are a peculiarity of HIV-specific responses is a question that is currently being studied in our laboratory. Chronic HIV infection is thought to induce higher proportions of Tregs as a mechanism preventing long-term damage caused by chronic immune activation [36]. On the other hand, these high levels of circulating Tregs could dampen Teffs responses and inadvertently help maintain viral persistence which, in turn, would lead to immune exhaustion. Therefore, the study of HIV-specific Tregs is a crucial aspect to consider in the quest for an efficient HIV-1 vaccine. The low levels suppression (30–35%) we obtained in our *in vitro* assays might not translate to what would have happened *in vivo* and more investigation using animal models would be more informative. Nevertheless, our point in this study was not to make a statement that the magnitude of Tregs’ suppression could be translated to a clinical impact but to show that these cells exert a suppressive effect.

When investigating whether vaccination shifted the balance of HIV-specific Tregs and Teffs, we found that the relative proportions of HIV-specific Tregs decreased significantly following vaccination. In contrast, Teffs increased in proportions, as measured by higher percentages of IFN-γ-, IL-2- and TNF-α- producing cells as well as increases in secretion of several other cytokines. Interestingly, the increase in these cytokines strongly correlated with the increase in LIPO-5-induced CD4^+^ specific responses. These results are in line with the fact that CD4^+^CD134^+^CD25^+^ antigen-specific cells contain several Th-subtype-defining transcription factors [25], and show that our vaccine indeed induced highly polyfunctional Th responses. In addition, we found that besides polyfunctionality, the breadth of the response is also an important predictive mark of vaccine effectiveness. Notably, patient N19, the only patient who did not experience viral rebound, responded strongly to all peptide pools after vaccination. These HIV-specific responses were not detected at entry prior to therapeutic immunization, thus suggesting that a shift to a less immunodominant response (such as the response to gag p17), could lead to a better distribution of the overall response and possibly a more effective viral control. This concept will be examined more in depth in our future trials. Of note, our vaccine contains palmitoyl-lysylamide lipid tail, known to signal through Toll-Like Receptor 2 and affect Tregs expansion and function in mouse studies [41,42]. Palmitoyl-lysylamide however may not have a similar role in human, as reflected by the decreased Tregs proportions observed after vaccination in our study.

An impact of HIV-specific Tregs on the elicited vaccine response was further supported by a consistent signal for an inverse correlation between both baseline and post-vaccination LIPO-5- specific Tregs, respectively, and post-vaccination immune scores. Although this did not reach statistical significance, as the analyses were likely underpowered due to the small sample size, these results suggest a negative role for Tregs in the induction of vaccine induced effector responses.

It would be of importance to know whether there is a clinical benefit in adding a Tregs blocker along with the vaccine in future studies. Outcomes from the cancer field clearly showed that Tregs suppress vaccine-induced immune responses and correlate with poor clinical benefit. In melanoma patients, reduction of suppressor cells by cyclophosphamide enhanced responses to vaccination [43]. Another study including patients with human papillomavirus type 16 (HPV16)-induced vulvar intraepithelial neoplasia, clearly showed that those with larger lesions mounted higher frequencies of HPV16-specific CD4^+^CD25^+^Foxp3^+^ T cells and displayed a lower HPV16-specific IFNγ/IL-10 ratio after vaccination [37], suggesting that high frequency of antigen-specific Tregs is predictive of poor clinical benefit. To circumvent the potential side-effects Tregs blocker could have on non-targeted immune responses, dendritic-cell based vaccination offers an interesting alternative. Pen *et al*. recently reported that multifunctional T cells could be induced without the induction of Tregs by vaccination with dendritic cells in which soluble PD1 or PD-L1 were induced by mRNA electroporation [44]. Also, with the future discovery of novel markers, we will be able to address the question of central *versus* peripheral origin of HIV-specific Tregs which could facilitate the *in vivo* targeting of these cells.

Another question that remains to be answered is whether effector specific-responses measured in patients after vaccination, were induced by naïve T cells priming or whether they originated from the preexisting pool of memory T cells. Although probably both priming of naïve cells and expansion of memory pool took place, we would need to use animal models to be able to track precursors and clearly address this question.

In addition, agonistic OX40 signaling itself could represent a good candidate for modulating vaccine responses towards a Th1 or Tregs in viral infections or autoimmune settings respectively. It was shown that when DCs were pulsed with KLH and injected to mice together with an anti-OX40 antibody, there was an increase in Th1 responses. In re-challenge experiments, OX40 stimulation led to the amplification of preexisting memory responses. These data suggest that skewing of the response based on OX40 ligation might be achieved only in unexposed individuals [23]. Of note, these findings need to be taken with caution as OX40, unlike in humans, is constitutively expressed on murine Tregs. Therefore, the modulation of the response by OX40 ligation in human and mouse is probably very different and needs further study. Nevertheless, this molecule may be an interesting target for future immunomodulation protocols, not only in HIV infection, but also in cancer and autoimmune settings.

In conclusion, we show here that the vaccination with DC-based vaccine pulsed with LIPO-5 construct, induced strong polyfunctional and polyspecific CD4^+^ T-cell responses. The strength of the induced responses inversely correlated with maximum viral load after antiretroviral treatment interruption. Importantly, the fact that we were able to measure Tregs and Teffs-specific cells in a single readout, gives our approach a significant advantage over other described approaches addressing the induction of CD4^+^ T-cell responses of different functional properties, especially in clinical trial settings.

## Materials and Methods

### Blood samples and vaccination procedure

Peripheral blood mononuclear cells (PBMCs) were obtained from healthy volunteers or vaccinees. Blood was collected in either heparin tubes or after apheresis. PBMCs were isolated from blood preparations by Ficoll density gradient centrifugation. All experiments were performed on freshly thawed cells that were left to rest for 5–6 hours in human serum-supplemented medium at 37°C.

ANRS/VRI DALIA 1, a phase I single-center study was performed at the North Texas Infectious Diseases Consultants in Dallas, TX. The study was sponsored by Baylor Institute for Immunology Research (BIIR) and the Agence Nationale de Recherches sur le SIDA et les hépatites virales (ANRS). DC-based vaccines were generated from blood monocytes by culturing with GM-CSF and IFN-α and additionally activated with LPS, as previously described [45]. Briefly, monocytes were obtained from the apheresis product of HAART-treated HIV-infected patients by elutriation and cultured in a closed system with GM-CSF/IFN-α for 3 days. Differentiating DCs were pulsed for the last 24 hours with the ANRS HIV LIPO-5 peptides: gag (17–35; 253–284); pol (325–355); and nef (66–97; 116–145). DCs were then activated with LPS (purified lipopolysaccharide prepared from Escherichia coli O:113; U.S. Standard Reference Endotoxin vialed under Good Manufacturing Practice guidelines) for 6 hours, harvested and frozen in autologous serum with a final concentration of 10% DMSO. After thawing, the DC vaccine cells suspended in 1 ml of freezing solution were diluted with 9 ml of saline to give a total volume for injection of 10 ml. Approximately 15x10^6^ viable frozen-thawed HIV lipopeptide-loaded DCs were injected subcutaneously in 3 separate injection sites (3.3 ml per site) in the upper and lower extremities. Subsequent DC injections were rotated to different locations on the upper and lower extremities. The vaccine was administered 4 times, at 4-weekly intervals. The blood samples (apheresis) analyzed were from wk -4, corresponding to the blood draw 4 weeks prior to first vaccination and wk 16, corresponding to the blood draw 4 weeks after the last vaccine. Antiviral treatment was stopped at wk 24 and viral load was measured thereafter.

### Ethics statement

Ethical committee approval and written informed consent from all subjects, in accordance with the Declaration of Helsinki, were obtained prior to study initiation. Committee and institutional review board(s) of EFS and INSERM (REF: C CPSL UNT—N° 12/EFS/079 and Convention reference number: I/DAJ/C2675) approved our study.

The study was approved by the IRB of Baylor Research Institute (BRI) (Clinical Trials Registration Number NCT 00796770). All patients gave written informed consent.

### Staining and phenotyping

All staining experiments were performed at 4°C for 30 minutes. Antibodies used were CD3-PerCPCy5.5, CD8-APCCy7, CD25-APC, CD134-PE, TNF-α-PECy7, CD154-APC ((Becton Dickinson (BD) Biosciences)), CD4-Alexa Fluor 700, IFN-γ-eFluor450, IL2-PerCPeFluor710, Streptavidin-Alexa Fluor 700 (eBioscience), FoxP3-Alexa Fluor 488, CD25-Brilliant Violet 421 (BioLegend), CD39-biotin, CD127-PE (Miltenyi biotec), Streptavidin-ECD, CD45RO-ECD (Beckman Coulter). LIVE/DEAD fixable aqua staining kit (Life technologies) was used to discriminate live and dead cells. For intracellular staining, FoxP3 buffer set (eBioscience) was used.

### T-cell sorting and functional assays

The “OX40 assay” is described in details elsewhere [21,22]. Briefly, two million PBMCs or Tregs-depleted cells were plated in 24-well plate and stimulated with 1μg/mL CMV lysate (Behring) or 2μg/mL of LIPO-5 or HIV peptide pools (192 peptides contained in 18 pools of 15-mers peptides (NeoMPS, Strasbourg, France) covering HIV-1 gag (G1 to G11 including 3 pools covering gag p17, 5 pools covering p24 and 3 pools gag p2/p6/p7), 4 pools of pol (RT12 to RT15) and 3 pools of nef (N16 to N18)) for 44 hours. In the last 6 hours, 1μg/mL of Brefeldin A (Sigma) was added to block the secretion of IFN-γ, IL-2 and TNF-α. Cells were then collected and stained for subsequent analysis by flow cytometry (BD LSR II).

Tregs-depleted PBMCs were obtained after efficient depletion of CD25^+^ Tregs as described previously [31]. The method comprised labeling total PBMCs with anti-CD25 beads (Miltenyi biotech) and one passage over LS columns. Briefly, 10 to 20 million PBMCs were used in all experiments. Ten microliters of anti-CD25 beads were added per 10x10^6^ PBMCs resuspended in 90μL of cold MACS buffer. Cells were then incubated for 20 minutes at 4°C then washed with 2–3 mL of MACS buffer before their passage through an LS column which has been placed on a manual magnetic separator. Both flow-through (Tregs-depleted) and remaining (Tregs) fractions were collected for further analysis and functional studies.

Tregs obtained by the above method were used in suppression assays in Tregs:Tresp ratio of 1:2. Either Tregs or non-Tregs were mixed with responding cells and incubated overnight in the presence of 2μg/mL of gag p24 peptide pool, 1 μg/mL of αCD28 and αCD49d (both from BD biosciences) and 10μg/mL of Brefeldin A. Responding cells were discriminated from Tregs (or non-Tregs) by labeling with carboxyfluorescein succinimidyl ester (CFSE, Life technologies) at 0.025mM final concentration for 15 min at 37°C.

FACS sorting of CD25^hi^, CD25^int^ and CD25^lo^ fractions were performed using MoFlo (Beckman Coulter, Hialeah, FL, USA). These CD4^+^ T-cell populations were subsequently cultured with non-CD4^+^ T cells in 1:4 ratio to “reconstitute” the conditions as for a standard PBMC “OX40 assay”.

### Multiplex cytokine secretion

After 44 hours of stimulation with LIPO-5, 500μL of each supernatant was collected and frozen at -80°C. Cytokine secretion measurement for TGF-β1, TGF-β2, IL-17F, IL-17A, IL-21, IL-22, IL-27, IL-31, IFN-γ, IL-10, IL-12p40, IL-12p70, IL-13, sCD40L, IL-9, IL-1β, IL-2, IL-3, IL-4, IL-5, IL-6, IL-8, IP-10, MCP-1, MIP-1β and TNF-α was performed using Luminex multiplex bead-based technology and a Bio-Plex 200 instrument (BioRad), according to the manufacturer’s directions. Data were analyzed both in terms of fluorescence intensity (FI) and after transformation to concentration (pg/ml) by a 5-parameter logistic curve, according to the manufacturer’s directions.

### Statistical analysis

Analyses of differences between pre- and post-vaccination time points were done by Wilcoxon matched-pairs signed rank test. Correlations were assessed by Spearman correlation coefficients.

To summarize the immune response to vaccination across several immune markers we used a method for multivariate ordinal data based on U-scores. Allowing for ties between variables, a partial ordering of the individuals is established based on their multivariate immunogenicity data, and an immune U-score for each individual is calculated by the difference in the numbers of individuals with superior *versus* inferior orders [46]. With this method we calculated a multivariate immune score across the following immune markers, best reflecting those correlated with maximum viral load post-ATI in the core trial analyses [18]: Luminex IL2, IL13, IL21 and IFN-γ after LIPO-5 stimulations of PBMC and % of IL-2, IFN-γ and TNF-α among CD134^+^CD25^+^ after LIPO-5 stimulation.

Prism 5.0, version 5.0d, (GraphPad Software, Inc.) and SAS V9.2 (SAS Institute, Cary, NC, USA) were used for statistical analyses. P values were considered significant when < 0.05, without adjustment for multiple testing in this exploratory study.

## Supporting Information

S1 Fig CMV-specific responses remained unchanged after the vaccination. IFN-γ, TNF-α and IL-2 production among CMV-specific cells (CD134^+^CD25^+^) (n = 14). Data were analyzed by Wilcoxon matched-pairs signed rank test. *p < 0.05; **p < 0.01; ***p < 0.001.
(TIF)

S2 Fig CD134^+^CD25^+^ cells are CD154^+^. Cells were stimulated for 44 hours with CMV lysate and stained with CD25, CD134 and CD154 6 hours after addition of Monensin. CD25^+^CD134^+^ (blue histogram) and CD25^-^CD134^-^ (gray filled histogram) are overlaid in the graph showing the expression of CD154 by each population.
(TIF)

S3 Fig Increases in LIPO-5-specific cells are followed by the increases in production of several cytokines. (A) Cytokines measured in supernatants after the PBMC stimulation with LIPO-5 for 44 hours. (B) Correlations between relative increases in LIPO-5-specific cells (x-axis) and cytokines detected in the supernatants after the stimulation with LIPO-5 of the same patients (y-axis) are given. Spearman coefficient for each correlation is indicated (r) as well as p value.
(TIF)

S4 Fig The average viral load post-ATI and viral load at the end of the follow up are inversely correlated with increased LIPO-5 specific responses. (A) Correlation between the average rebound viral load and maximum viral load rebound after HAART interruption (n = 14). (B) Correlation between the relative increase in LIPO-5-specific response (response after the vaccination-response before vaccination) and average rebound viral load after HAART interruption (n = 14). (C) Correlation between the relative increase in LIPO-5-specific response (response after the vaccination-response before vaccination) and viral load at the end of ATI (n = 14). Spearman coefficient is indicated (r) as well as p value.
(TIF)

S5 Fig The amount of gag p24- specific CD4^+^ T cells inversely correlates with viral load rebound after ATI. Graph shows the correlation between the relative increase in gag p24-specific response (response after the vaccination-response before vaccination) and maximal viral load rebound after HAART interruption (n = 14). Spearman coefficient is indicated (r) as well as p value.
(TIF)

S6 Fig Antigen-specific CD4^+^ T cells (CD25^+^CD134^+^) *versus* CD25^+^CD134^-^ cells. Representative plots and gating strategy of viable CD4^+^ T cells after the stimulation with CMV lysate. (A) Antigen-specific cells (CD134^+^CD25^+^) secrete different cytokines (IFN-γ, TNF-α and IL-2), as well as express Tregs markers (FoxP3 and CD39). (B) CD4^+^ T cells expressing only CD25 after stimulation are Tregs. CD25^+^ cells, unlike CD25^+^CD134^+^ do not contain cytokine-secreting cells (IFN-γ, TNF-α and IL-2) and they express Tregs markers (FoxP3 and CD39).
(TIF)

S7 Fig CD39^+^ Tregs are Helios^+^. Representative plots showing Helios expression on FoxP3^+^CD39^+^ (red), FoxP3^+^CD39^-^ (blue) or FoxP3^-^CD39^-^ (orange) in bulk CD4^+^ T cells (upper panels) or CMV-specific CD4^+^CD134^+^CD25^+^ cells (lower panels).
(TIF)

S1 Table HIV-specific responses. Individual responses to each antigenic stimulation are given. Color code as indicated in the table reflects the strength of the response: weak response (%CD4^+^CD25^+^CD134^+^ <1%, in grey) (strength = 1), medium response (1%< %CD4^+^CD25^+^CD134^+^ <2%, in yellow) (strength = 2), strong response (2%< %CD4^+^CD25^+^CD134^+^ <3%, in orange) (strength = 3) and a very strong response (%CD4^+^CD25^+^CD134^+^ >3%, in red) (strength = 4).
(DOCX)

## References

[1] DAD Study Group, Friis-Møller N, Reiss P, Sabin CA, Weber R, et al (2007) Class of antiretroviral drugs and the risk of myocardial infarction. N Engl J Med 356: 1723–1735. 10.1056/NEJMoa062744 17460226

[2] Cazanave C, Dupon M, Lavignolle-Aurillac V, Barthe N, Lawson-Ayayi S, et al (2008) Reduced bone mineral density in HIV-infected patients: prevalence and associated factors. AIDS 22: 395–402. 10.1097/QAD.0b013e3282f423dd 18195566

[3] Bonnet F, Amieva H, Marquant F, Bernard C, Bruyand M, et al (2013) Cognitive disorders in HIV-infected patients: are they HIV-related? AIDS 27: 391–400. 10.1097/QAD.0b013e32835b1019 23079813

[4] McMichael AJ (2006) HIV vaccines. Annu Rev Immunol 24: 227–255. 10.1146/annurev.immunol.24.021605.090605 16551249

[5] Fauci AS, Johnston MI, Dieffenbach CW, Burton DR, Hammer SM, et al (2008) HIV vaccine research: the way forward. Science 321: 530–532. 10.1126/science.1161000 18653883

[6] Appay V (2009) 25 years of HIV research!… and what about a vaccine? Eur J Immunol 39: 1999–2003. 10.1002/eji.200939551 19672891

[7] Lévy Y, Gahéry-Ségard H, Durier C, Lascaux A-S, Goujard C, et al (2005) Immunological and virological efficacy of a therapeutic immunization combined with interleukin-2 in chronically HIV-1 infected patients. AIDS 19: 279–286. 15718838

[8] Lévy Y, Durier C, Lascaux A-S, Meiffrédy V, Gahéry-Ségard H, et al (2006) Sustained control of viremia following therapeutic immunization in chronically HIV-1-infected individuals. AIDS 20: 405–413. 10.1097/01.aids.0000206504.09159.d3 16439874

[9] Hansen SG, Vieville C, Whizin N, Coyne-Johnson L, Siess DC, et al (2009) Effector memory T cell responses are associated with protection of rhesus monkeys from mucosal simian immunodeficiency virus challenge. Nat Med 15: 293–299. 10.1038/nm.1935 19219024 PMC2720091

[10] Hansen SG, Ford JC, Lewis MS, Ventura AB, Hughes CM, et al (2011) Profound early control of highly pathogenic SIV by an effector memory T-cell vaccine. Nature 473: 523–527. 10.1038/nature10003 21562493 PMC3102768

[11] Banchereau J, Steinman RM (1998) Dendritic cells and the control of immunity. Nature 392: 245–252. 10.1038/32588 9521319

[12] Banchereau J, Briere F, Caux C, Davoust J, Lebecque S, et al (2000) Immunobiology of dendritic cells. Annu Rev Immunol 18: 767–811. 10.1146/annurev.immunol.18.1.767 10837075

[13] Palucka K, Banchereau J (2013) Human dendritic cell subsets in vaccination. Curr Opin Immunol 25: 396–402. 10.1016/j.coi.2013.05.001 23725656 PMC3711217

[14] García F, Routy J-P (2011) Challenges in dendritic cells-based therapeutic vaccination in HIV-1 infection Workshop in dendritic cell-based vaccine clinical trials in HIV-1. Vaccine 29: 6454–6463. 10.1016/j.vaccine.2011.07.043 21791232

[15] Lu W, Arraes LC, Ferreira WT, Andrieu J-M (2004) Therapeutic dendritic-cell vaccine for chronic HIV-1 infection. Nat Med 10: 1359–1365. 10.1038/nm1147 15568033

[16] Van Gulck E, Vlieghe E, Vekemans M, Van Tendeloo VFI, Van De Velde A, et al (2012) mRNA-based dendritic cell vaccination induces potent antiviral T-cell responses in HIV-1-infected patients. AIDS 26: F1–12. 10.1097/QAD.0b013e32834f33e8 22156965

[17] García F, Climent N, Guardo AC, Gil C, León A, et al (2013) A dendritic cell-based vaccine elicits T cell responses associated with control of HIV-1 replication. Sci Transl Med 5: 166ra2 10.1126/scitranslmed.3004682 23283367

[18] Lévy Y, Thiébaut R, Montes M, Lacabaratz C, Sloan L, et al (2014) Dendritic cell-based therapeutic vaccine elicits polyfunctional HIV-specific T-cell immunity associated with control of viral load. Eur J Immunol 44: 2802–2810. 10.1002/eji.201344433 25042008

[19] Weiss L, Donkova-Petrini V, Caccavelli L, Balbo M, Carbonneil C, et al (2004) Human immunodeficiency virus-driven expansion of CD4+CD25+ regulatory T cells, which suppress HIV-specific CD4 T-cell responses in HIV-infected patients. Blood 104: 3249–3256. 10.1182/blood-2004-01-0365 15271794

[20] Takahashi T, Kuniyasu Y, Toda M, Sakaguchi N, Itoh M, et al (1998) Immunologic self-tolerance maintained by CD25+CD4+ naturally anergic and suppressive T cells: induction of autoimmune disease by breaking their anergic/suppressive state. Int Immunol 10: 1969–1980. 9885918 10.1093/intimm/10.12.1969

[21] Zaunders JJ, Munier ML, Seddiki N, Pett S, Ip S, et al (2009) High levels of human antigen-specific CD4+ T cells in peripheral blood revealed by stimulated coexpression of CD25 and CD134 (OX40). J Immunol 183: 2827–2836. 10.4049/jimmunol.0803548 19635903

[22] Seddiki N, Cook L, Hsu DC, Phetsouphanh C, Brown K, et al (2014) Human antigen-specific CD4+CD25+CD134+CD39+ T cells are enriched for regulatory T cells and comprise a substantial proportion of recall responses. Eur J Immunol: n/a–n/a. 10.1002/eji.201344102 24752698

[23] Croft M (2010) Control of Immunity by the TNFR-Related Molecule OX40 (CD134). Annu Rev Immunol 28: 57–78. 10.1146/annurev-immunol-030409-101243 20307208 PMC2882161

[24] Chattopadhyay PK, Yu J, Roederer M (2006) Live-cell assay to detect antigen-specific CD4+ T-cell responses by CD154 expression. Nat Protocols 1: 1–6. 10.1038/nprot.2006.1 17406204

[25] Phetsouphanh C, Xu Y, Amin J, Seddiki N, Procopio F, et al (2013) Characterization of transcription factor phenotypes within antigen-specific CD4+ T cells using qualitative multiplex single-cell RT-PCR. PLoS ONE 8: e74946 10.1371/journal.pone.0074946 24124462 PMC3790772

[26] Thornton AM, Korty PE, Tran DQ, Wohlfert EA, Murray PE, et al (2010) Expression of Helios, an Ikaros transcription factor family member, differentiates thymic-derived from peripherally induced Foxp3+ T regulatory cells. J Immunol 184: 3433–3441. 10.4049/jimmunol.0904028 20181882 PMC3725574

[27] Himmel ME, MacDonald KG, Garcia RV, Steiner TS, Levings MK (2013) Helios+ and Helios- cells coexist within the natural FOXP3+ T regulatory cell subset in humans. J Immunol 190: 2001–2008. 10.4049/jimmunol.1201379 23359504

[28] Allan SE, Crome SQ, Crellin NK, Passerini L, Steiner TS, et al (2007) Activation-induced FOXP3 in human T effector cells does not suppress proliferation or cytokine production. Int Immunol 19: 345–354. 10.1093/intimm/dxm014 17329235

[29] Miyara M, Yoshioka Y, Kitoh A, Shima T, Wing K, et al (2009) Functional delineation and differentiation dynamics of human CD4+ T cells expressing the FoxP3 transcription factor. Immunity 30: 899–911. 10.1016/j.immuni.2009.03.019 19464196

[30] McMurchy AN, Levings MK (2012) Suppression assays with human T regulatory cells: a technical guide. Eur J Immunol 42: 27–34. 10.1002/eji.201141651 22161814

[31] Brezar V, Ruffin N, Lévy Y, Seddiki N (2014) A highly relevant and efficient single step method for simultaneous depletion and isolation of human regulatory T cells in a clinical setting. J Immunol Methods. 10.1016/j.jim.2014.06.003 24925808

[32] Maeda Y, Nishikawa H, Sugiyama D, Ha D, Hamaguchi M, et al (2014) Detection of self-reactive CD8^+^ T cells with an anergic phenotype in healthy individuals. Science 346: 1536–1540. 10.1126/science.aaa1292 25525252

[33] Chevalier MF, Jülg B, Pyo A, Flanders M, Ranasinghe S, et al (2011) HIV-1-specific interleukin-21+ CD4+ T cell responses contribute to durable viral control through the modulation of HIV-specific CD8+ T cell function. J Virol 85: 733–741. 10.1128/JVI.02030-10 21047960 PMC3020027

[34] Norris PJ, Moffett HF, Yang OO, Kaufmann DE, Clark MJ, et al (2004) Beyond help: direct effector functions of human immunodeficiency virus type 1-specific CD4(+) T cells. J Virol 78: 8844–8851. 10.1128/JVI.78.16.8844-8851.2004 15280492 PMC479080

[35] Stockinger B, Bourgeois C, Kassiotis G (2006) CD4+ memory T cells: functional differentiation and homeostasis. Immunol Rev 211: 39–48. 10.1111/j.0105-2896.2006.00381 16824115

[36] Seddiki N, Brezar V, Draenert R (2014) Cell exhaustion in HIV-1 infection: role of suppressor cells. Curr Opin HIV AIDS. 10.1097/COH.0000000000000087 25010895

[37] Welters MJP, Kenter GG, De Vos van Steenwijk PJ, Löwik MJG, Berends-van der Meer DMA, et al (2010) Success or failure of vaccination for HPV16-positive vulvar lesions correlates with kinetics and phenotype of induced T-cell responses. Proc Natl Acad Sci USA 107: 11895–11899. 10.1073/pnas.1006500107 20547850 PMC2900675

[38] Sugiyama D, Nishikawa H, Maeda Y, Nishioka M, Tanemura A, et al (2013) Anti-CCR4 mAb selectively depletes effector-type FoxP3+CD4+ regulatory T cells, evoking antitumor immune responses in humans. Proc Natl Acad Sci USA 110: 17945–17950. 10.1073/pnas.1316796110 24127572 PMC3816454

[39] Macatangay BJC, Szajnik ME, Whiteside TL, Riddler SA, Rinaldo CR (2010) Regulatory T cell suppression of Gag-specific CD8 T cell polyfunctional response after therapeutic vaccination of HIV-1-infected patients on ART. PLoS ONE 5: e9852 10.1371/journal.pone.0009852 20352042 PMC2844424

[40] Angin M, King M, Altfeld M, Walker BD, Wucherpfennig KW, et al (2012) Identification of HIV-1-specific regulatory T-cells using HLA class II tetramers. AIDS 26: 2112–2115. 10.1097/QAD.0b013e328358cc75 22874519 PMC3825094

[41] Sutmuller RPM, Den Brok MHMGM, Kramer M, Bennink EJ, Toonen LWJ, et al (2006) Toll-like receptor 2 controls expansion and function of regulatory T cells. J Clin Invest 116: 485–494. 10.1172/JCI25439 16424940 PMC1332026

[42] Liu H, Komai-Koma M, Xu D, Liew FY (2006) Toll-like receptor 2 signaling modulates the functions of CD4+ CD25+ regulatory T cells. Proc Natl Acad Sci USA 103: 7048–7053. 10.1073/pnas.0601554103 16632602 PMC1444884

[43] Sierro SR, Donda A, Perret R, Guillaume P, Yagita H, et al (2011) Combination of lentivector immunization and low-dose chemotherapy or PD-1/PD-L1 blocking primes self-reactive T cells and induces anti-tumor immunity. Eur J Immunol 41: 2217–2228. 10.1002/eji.201041235 21538347

[44] Pen JJ, Keersmaecker BD, Heirman C, Corthals J, Liechtenstein T, et al (2014) Interference with PD-L1/PD-1 co-stimulation during antigen presentation enhances the multifunctionality of antigen-specific T cells. Gene Ther 21: 262–271. 10.1038/gt.2013.80 24401835

[45] Cobb A, Roberts LK, Palucka AK, Mead H, Montes M, et al (2011) Development of a HIV-1 lipopeptide antigen pulsed therapeutic dendritic cell vaccine. J Immunol Methods 365: 27–37. 10.1016/j.jim.2010.11.002 21093448

[46] Wittkowski KM, Lee E, Nussbaum R, Chamian FN, Krueger JG (2004) Combining several ordinal measures in clinical studies. Stat Med 23: 1579–1592. 10.1002/sim.1778 15122738

