# Supplementary figures and images for: Decreased HIV-Specific T-Regulatory Responses Are Associated with Effective DC-Vaccine Induced Immunity
Source: PLoS Pathog. 2015 Mar 27;11(3):e1004752. doi: 10.1371/journal.ppat.1004752 (PMC4376642; doi:10.1371/journal.ppat.1004752)

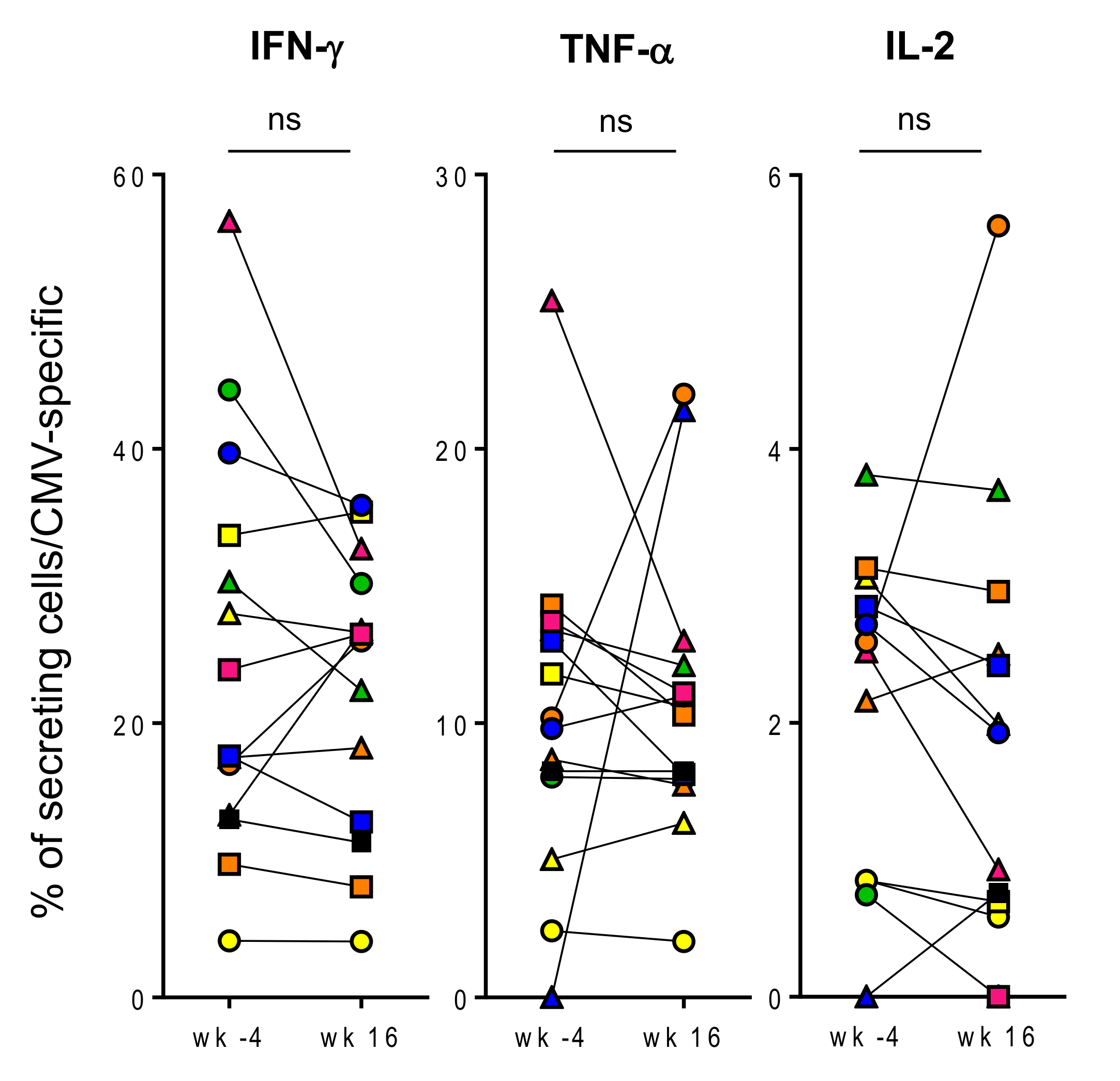

Supplement: S1 Fig — IFN-γ, TNF-α and IL-2 production among CMV-specific cells (CD134+CD25+) (n = 14). Data were analyzed by Wilcoxon matched-pairs signed rank test. *p < 0.05; **p < 0.01; ***p < 0.001. (TIF) [file ppat.1004752.s001.tif]

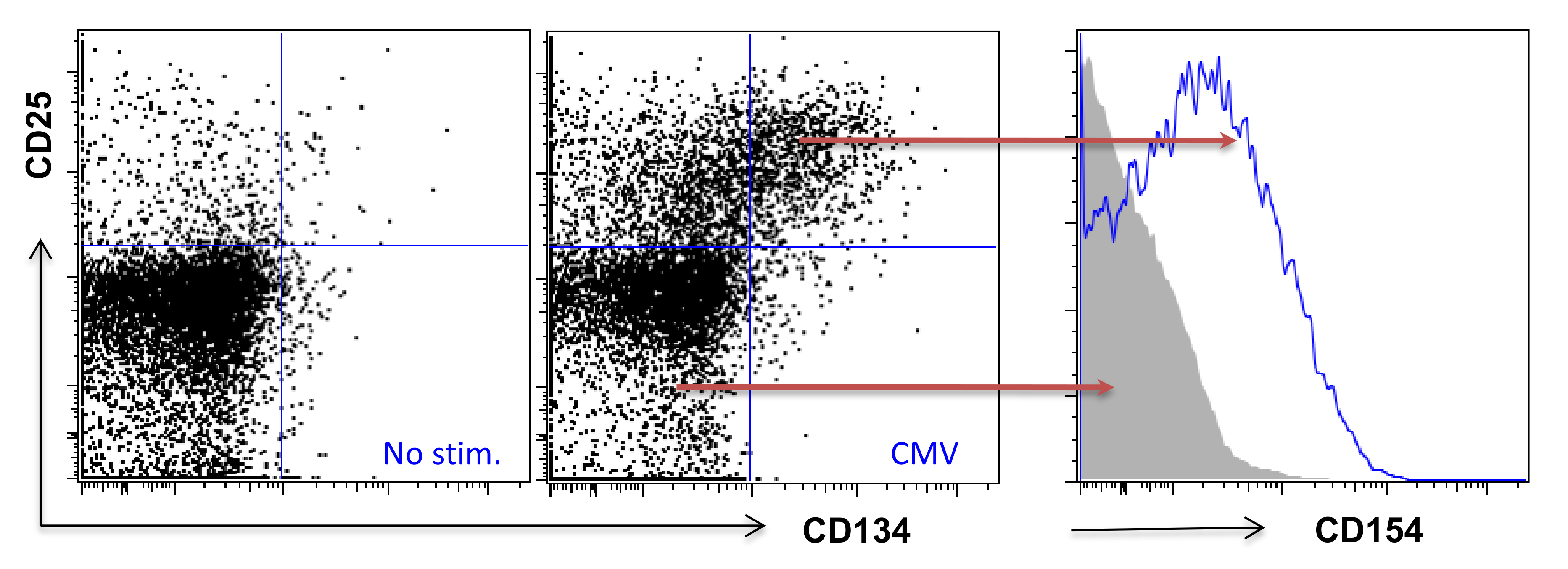

Supplement: S2 Fig — Cells were stimulated for 44 hours with CMV lysate and stained with CD25, CD134 and CD154 6 hours after addition of Monensin. CD25+CD134+ (blue histogram) and CD25-CD134- (gray filled histogram) are overlaid in the graph showing the expression of CD154 by each population. (TIF) [file ppat.1004752.s002.tif]

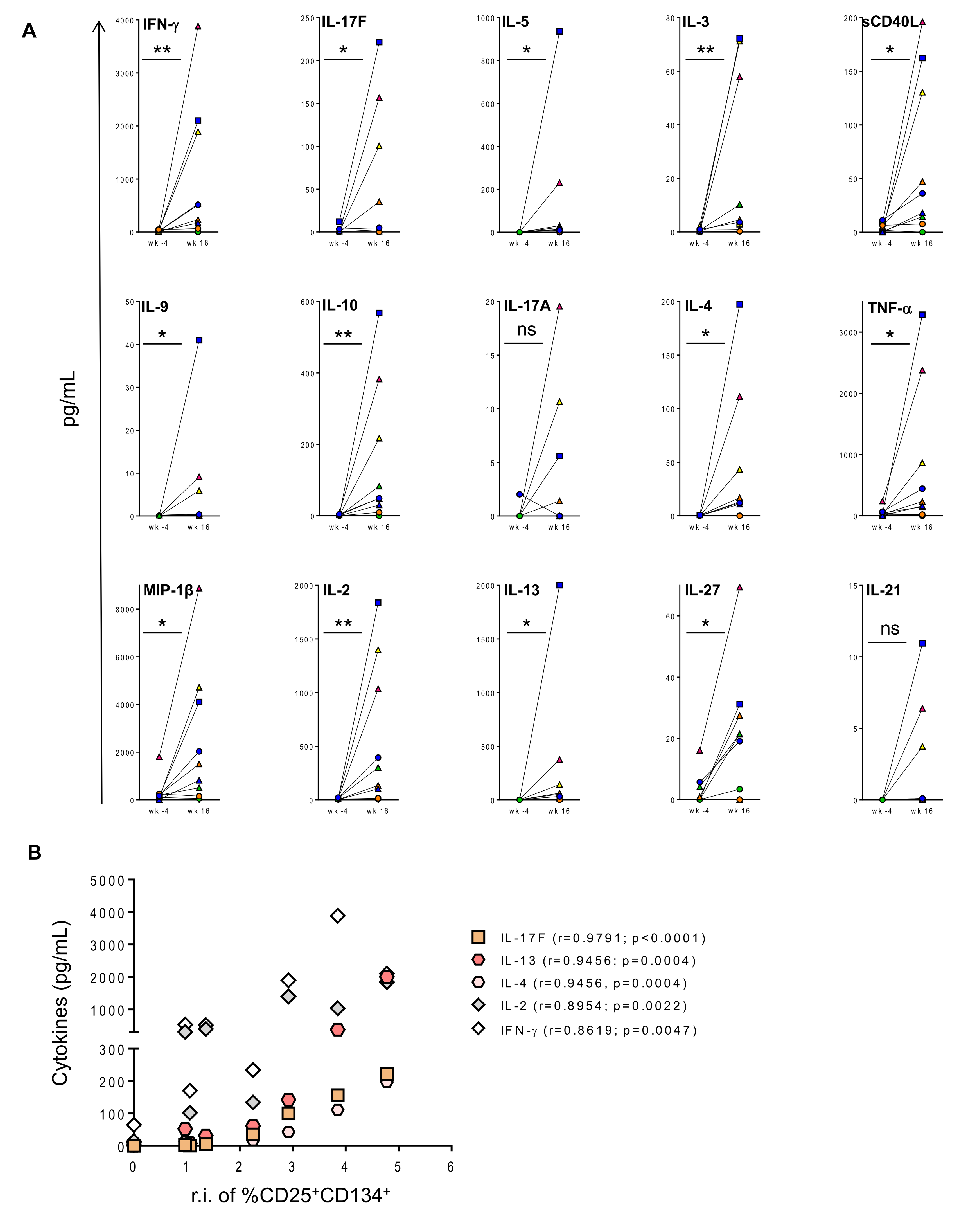

Supplement: S3 Fig — (A) Cytokines measured in supernatants after the PBMC stimulation with LIPO-5 for 44 hours. (B) Correlations between relative increases in LIPO-5-specific cells (x-axis) and cytokines detected in the supernatants after the stimulation with LIPO-5 of the same patients (y-axis) are given. Spearman coefficient for each correlation is indicated (r) as well as p value. (TIF) [file ppat.1004752.s003.tif]

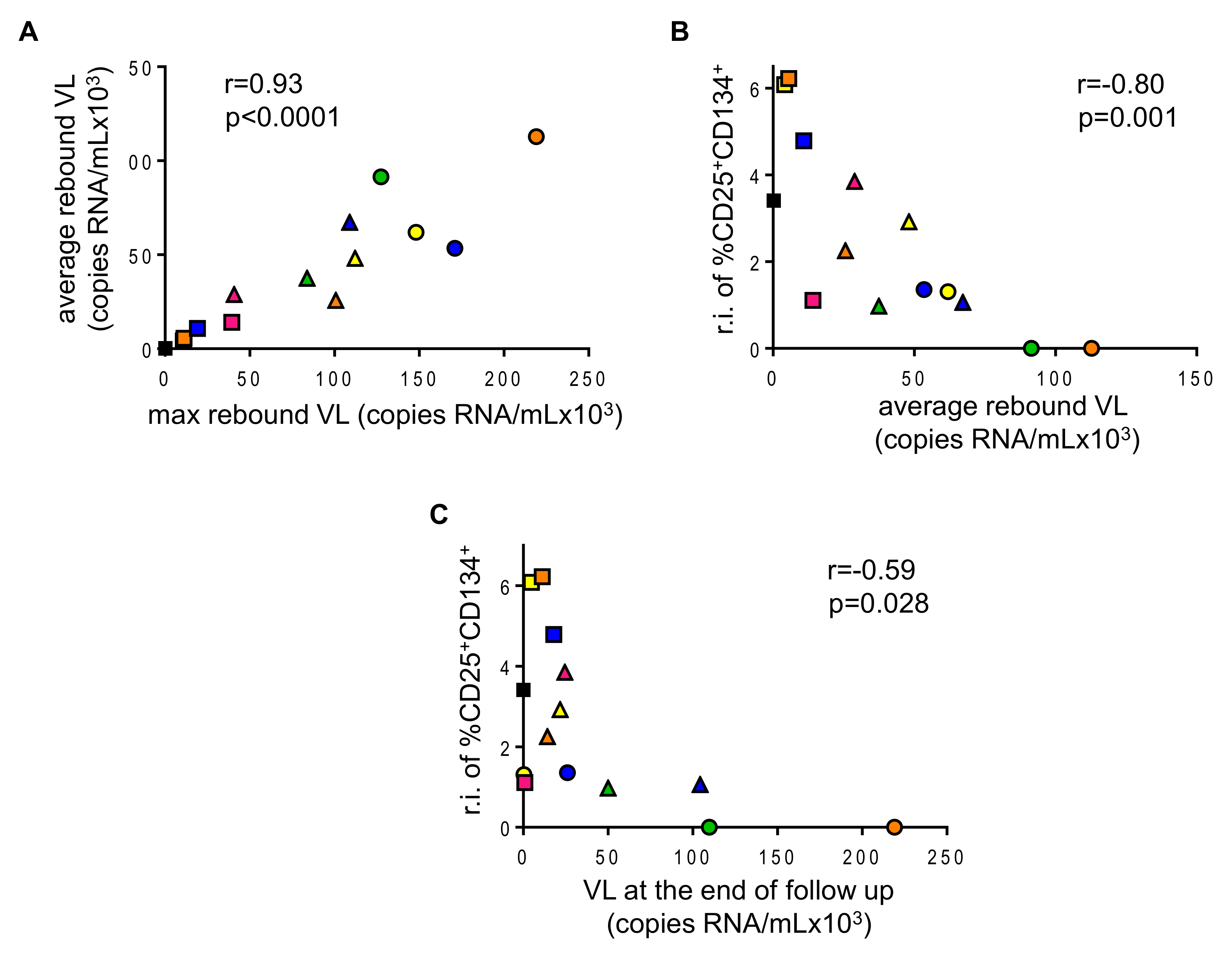

Supplement: S4 Fig — (A) Correlation between the average rebound viral load and maximum viral load rebound after HAART interruption (n = 14). (B) Correlation between the relative increase in LIPO-5-specific response (response after the vaccination-response before vaccination) and average rebound viral load after HAART interruption (n = 14). (C) Correlation between the relative increase in LIPO-5-specific response (response after the vaccination-response before vaccination) and viral load at the end of ATI (n = 14). Spearman coefficient is indicated (r) as well as p value. (TIF) [file ppat.1004752.s004.tif]

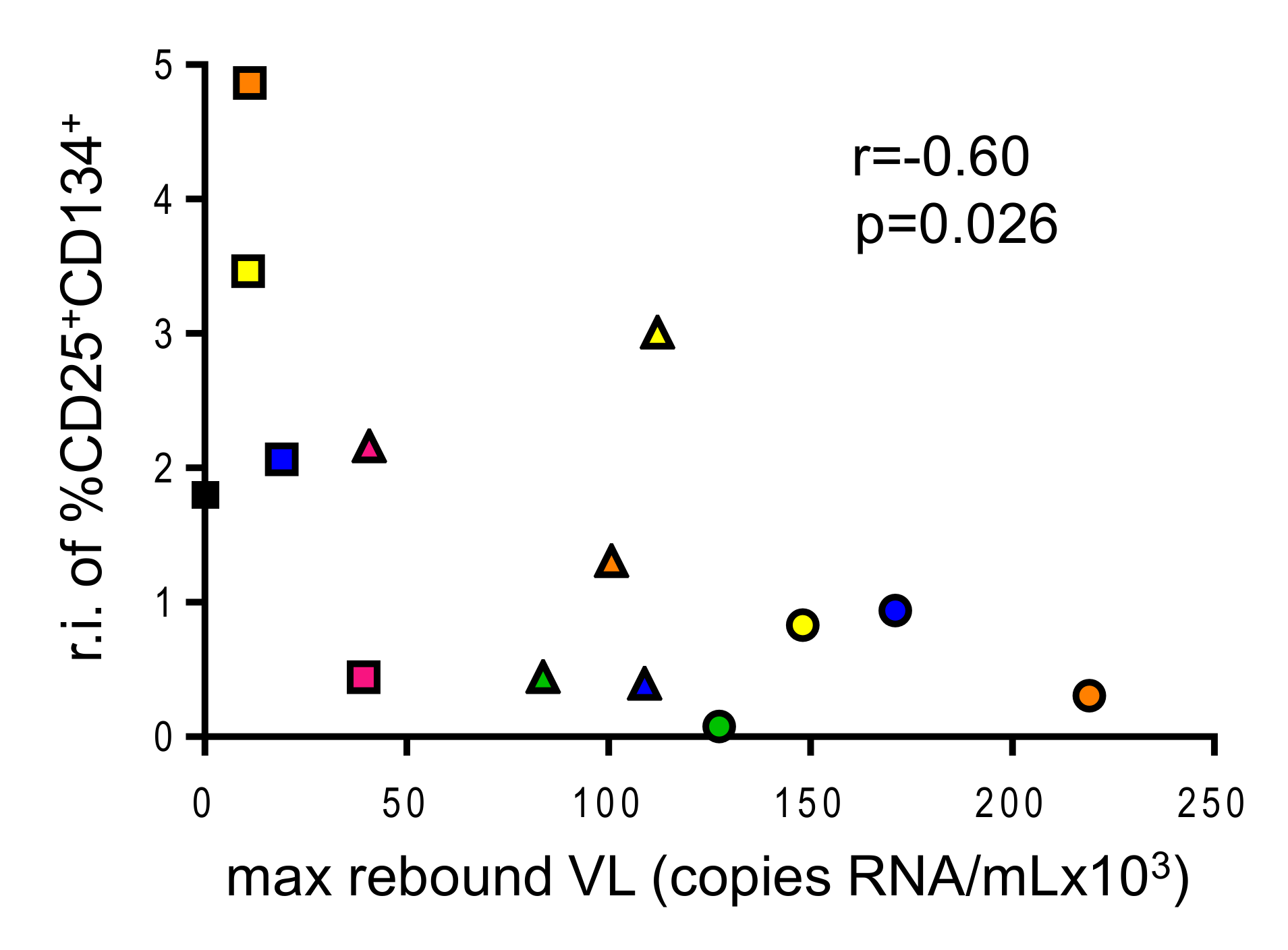

Supplement: S5 Fig — Graph shows the correlation between the relative increase in gag p24-specific response (response after the vaccination-response before vaccination) and maximal viral load rebound after HAART interruption (n = 14). Spearman coefficient is indicated (r) as well as p value. (TIF) [file ppat.1004752.s005.tif]

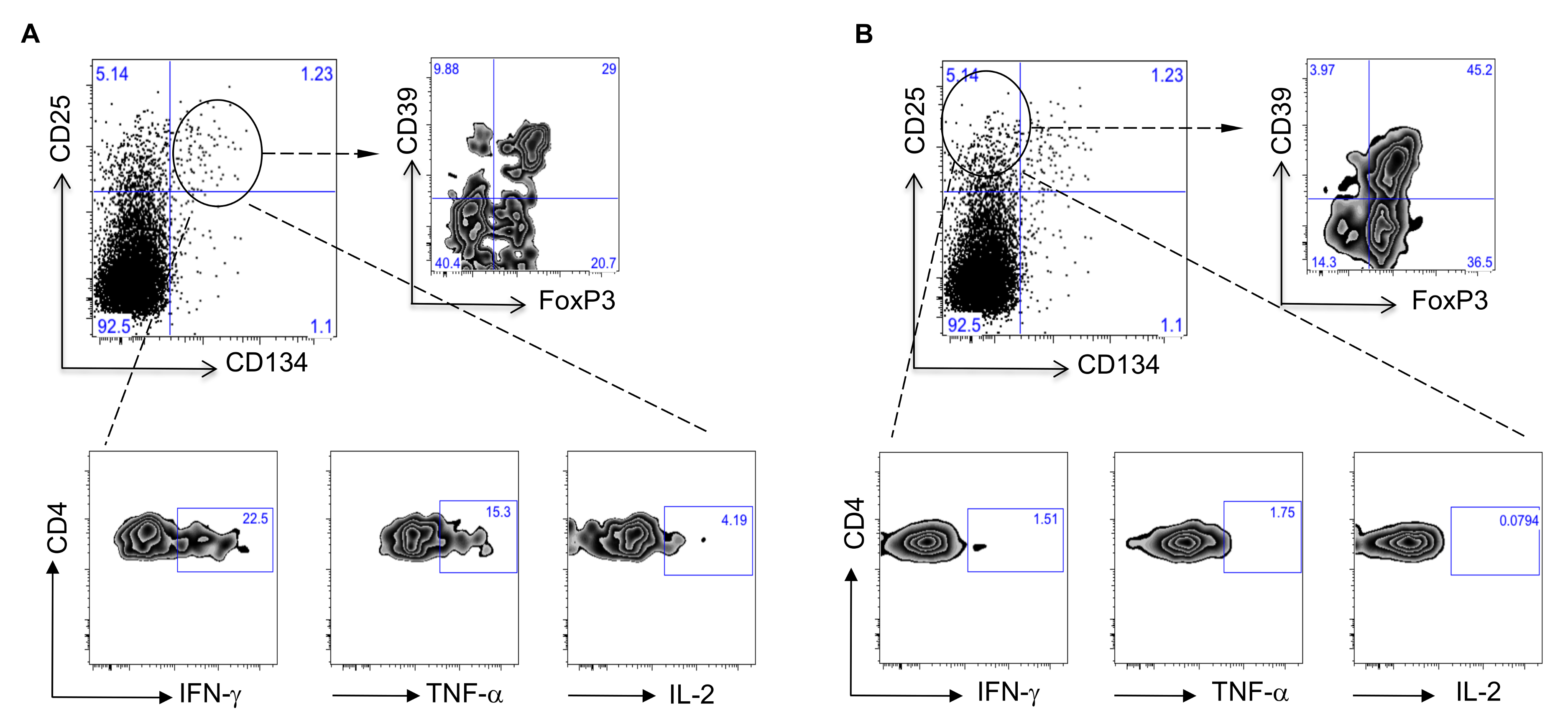

Supplement: S6 Fig — Representative plots and gating strategy of viable CD4+ T cells after the stimulation with CMV lysate. (A) Antigen-specific cells (CD134+CD25+) secrete different cytokines (IFN-γ, TNF-α and IL-2), as well as express Tregs markers (FoxP3 and CD39). (B) CD4+ T cells expressing only CD25 after stimulation are Tregs. CD25+ cells, unlike CD25+CD134+ do not contain cytokine-secreting cells (IFN-γ, TNF-α and IL-2) and they express Tregs markers (FoxP3 and CD39). (TIF) [file ppat.1004752.s006.tif]

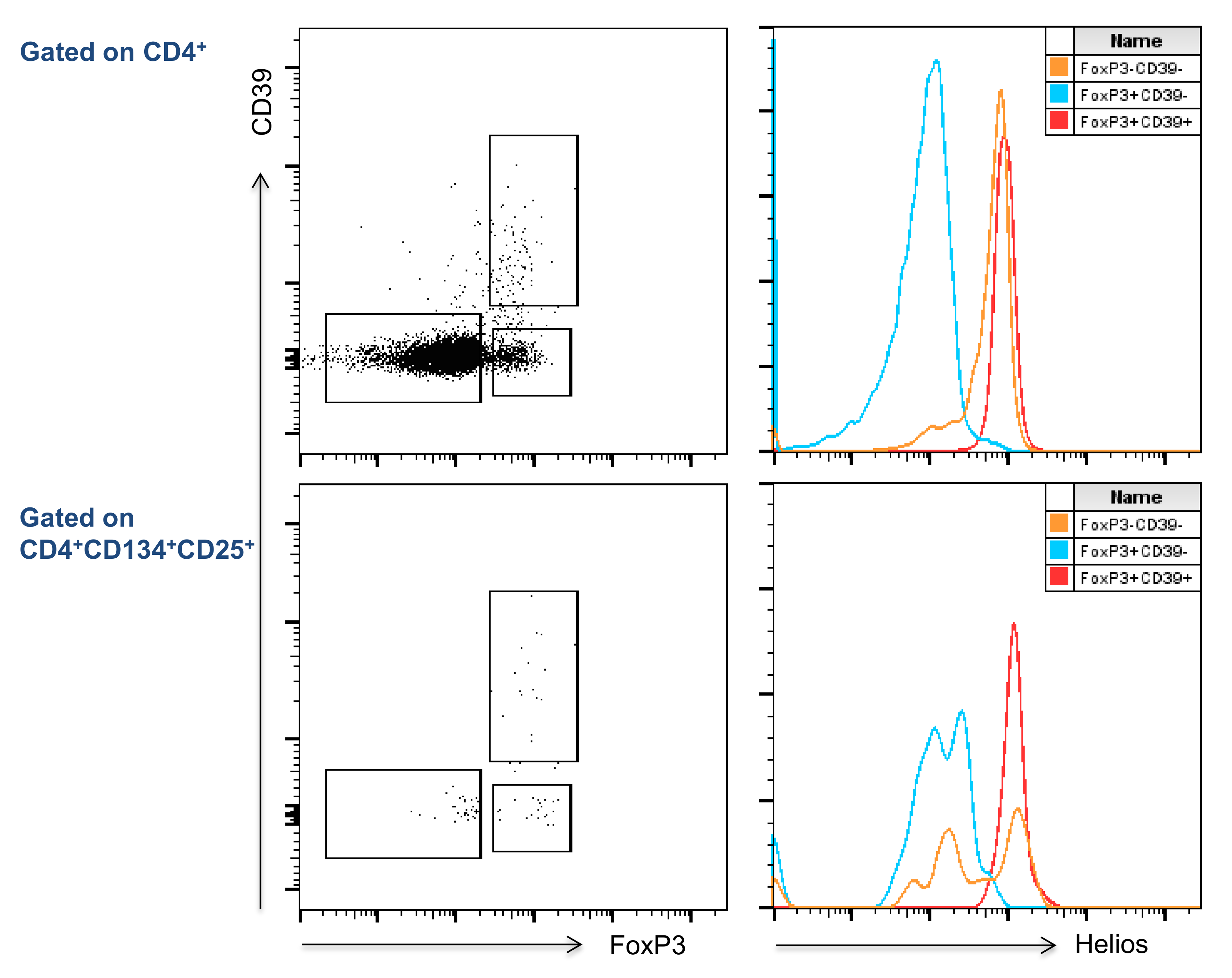

Supplement: S7 Fig — Representative plots showing Helios expression on FoxP3+CD39+ (red), FoxP3+CD39- (blue) or FoxP3-CD39- (orange) in bulk CD4+ T cells (upper panels) or CMV-specific CD4+CD134+CD25+ cells (lower panels). (TIF) [file ppat.1004752.s007.tif]
