# Supplementary material for: Decreased HIV-Specific T-Regulatory Responses Are Associated with Effective DC-Vaccine Induced Immunity
Source: PLoS Pathog. 2015 Mar 27;11(3):e1004752. doi: 10.1371/journal.ppat.1004752 (PMC4376642; doi:10.1371/journal.ppat.1004752)
Supplement: S1 Table — Individual responses to each antigenic stimulation are given. Color code as indicated in the table reflects the strength of the response: weak response (%CD4+CD25+CD134+ <1%, in grey) (strength = 1), medium response (1%< %CD4+CD25+CD134+ <2%, in yellow) (strength = 2), strong response (2%< %CD4+CD25+CD134+ <3%, in orange) (strength = 3) and a very strong response (%CD4+CD25+CD134+ >3%, in red) (strength = 4). (DOCX) [file ppat.1004752.s008.docx]

| **Patient** | **Week** | **LIPO-5** | **gag p24** | **gag p2-6** | **gag p17** | **pol** | **nef** |
| --- | --- | --- | --- | --- | --- | --- | --- |
| **1**  **(yellow circle)** | -4 | 0 | 1.12 | - | - | - | - |
|  | 16 | 1.31 | 1.95 | - | - | - | - |
| **2**  **(green circle)** | -4 | 0.0863 | 0.258 | - | - | - | - |
|  | 16 | 0 | 0.337 | - | - | - | - |
| **3**  **(yellow square)** | -4 | 0 | 0.257 | 0.203 | 0.171 | 0.3 | 0 |
|  | 16 | 6.08 | 3.72 | 0.274 | 0.856 | 4.64 | 0.666 |
| **4**  **(yellow triangle)** | -4 | 0.163 | 0.772 | - | - | - | - |
|  | 16 | 3.08 | 3.78 | - | - | - | - |
| **5**  **(green triangle)** | -4 | 0.41 | 0.877 | - | - | - | - |
|  | 16 | 1.38 | 1.32 | - | - | - | - |
| **6**  **(orange triangle)** | -4 | 0.16 | 0.332 | - | - | - | - |
|  | 16 | 2.41 | 1.64 | - | - | - | - |
| **7**  **(pink triangle)** | -4 | 0 | 0 | 0 | 0 | 0 | 0 |
|  | 16 | 3.85 | 2.16 | 0 | 0.6 | 2.82 | 2.59 |
| **8**  **(blue triangle)** | -4 | 0 | 0 | 0 | 0 | 0 | 0 |
|  | 16 | 1.06 | 0.394 | 0 | 0.132 | 0.654 | 0.202 |
| **9**  **(orange square)** | -4 | 0.308 | 0.595 | 0.146 | 0.108 | 0.329 | 1.38 |
|  | 16 | 6.53 | 5.46 | 0 | 1.75 | 1.64 | 1.67 |
| **10**  **(orange circle)** | -4 | 1.57 | 0 | 0 | 0 | 0 | 1.28 |
|  | 16 | 0.775 | 0.308 | 0 | 0 | 0 | 0.683 |
| **11**  **(blue square)** | -4 | 0.106 | 0.295 | 0.126 | 0.15 | 0 | 0 |
|  | 16 | 4.89 | 2.36 | 0.689 | 2.03 | 1.59 | 0.6 |
| **14**  **(blue circle)** | -4 | 0.465 | 1.09 | 0.27 | 0.313 | 0.214 | 0.153 |
|  | 16 | 1.82 | 2.03 | 0.407 | 0.874 | 0.303 | 0.783 |
| **18**  **(pink square)** | -4 | 0 | 0.235 | 0 | 0 | 0 | 0 |
|  | 16 | 1.11 | 0.677 | 0 | 0.18 | 0.396 | 0.628 |
| **19**  **(black square)** | -4 | 0 | 0 | 0 | 0 | 0 | 0 |
|  | 16 | 3.41 | 1.8 | 0 | 3.03 | 1.59 | 2.42 |

**Strength 1**:

weak response <1%

**Strength 2**: response

between 1% and 2%

**Strength 3**: response

between 2% and 3%

**Strength 4**:

response higher than 3%
